# Spatial dissection of the *Arabidopsis thaliana* transcriptional response to downy mildew using Fluorescence Activated Cell Sorting

**DOI:** 10.3389/fpls.2015.00527

**Published:** 2015-07-10

**Authors:** Timothy L. R. Coker, Volkan Cevik, Jim L. Beynon, Miriam L. Gifford

**Affiliations:** ^1^Systems Biology Doctoral Training Centre, University of WarwickCoventry, UK; ^2^School of Life Sciences, University of WarwickCoventry, UK

**Keywords:** plant-pathogen interactions, oomycete pathogens, biotrophic infection, cell type-specific transcriptomics, Fluorescence Activated Cell Sorting

## Abstract

Changes in gene expression form a crucial part of the plant response to infection. In the last decade, whole-leaf expression profiling has played a valuable role in identifying genes and processes that contribute to the interactions between the model plant *Arabidopsis thaliana* and a diverse range of pathogens. However, with some pathogens such as downy mildew caused by the biotrophic oomycete pathogen *Hyaloperonospora arabidopsidis* (*Hpa*), whole-leaf profiling may fail to capture the complete *Arabidopsis* response encompassing responses of non-infected as well as infected cells within the leaf. Highly localized expression changes that occur in infected cells may be diluted by the comparative abundance of non-infected cells. Furthermore, local and systemic *Hpa* responses of a differing nature may become conflated. To address this we applied the technique of Fluorescence Activated Cell Sorting (FACS), typically used for analyzing plant abiotic responses, to the study of plant-pathogen interactions. We isolated haustoriated (*Hpa*-proximal) and non-haustoriated (*Hpa*-distal) cells from infected seedling samples using FACS, and measured global gene expression. When compared with an uninfected control, 278 transcripts were identified as significantly differentially expressed, the vast majority of which were differentially expressed specifically in *Hpa*-proximal cells. By comparing our data to previous, whole organ studies, we discovered many highly locally regulated genes that can be implicated as novel in the *Hpa* response, and that were uncovered for the first time using our sensitive FACS technique.

## Introduction

Unlike mammals, plants do not develop specialized immune cells. Instead, they rely on Pattern-Recognition Receptors (PRRs), which detect conserved molecules or motifs associated with foreign micro-organisms (Zipfel, 2014), and cytoplasmic NOD-Like Receptors (NLRs), which detect more specific pathogen-derived effectors that are delivered into the plant cell (Jones and Dangl, 2006). Perception of a pathogen by these receptors triggers a cascade of cellular signaling events, which culminate at the cell nucleus where transcriptional reprogramming occurs (Tsuda and Somssich, 2015).

Transcriptional reprogramming is a crucial part of the immune response, and this makes it a potential target for interference from pathogens. Manipulation of host gene expression may be particularly important for biotrophic pathogens, which must keep their host cells alive while effectively suppressing the immune system and extracting nutrients. A number of pathogenic effectors from *Pseudomonas syringae* and *Hyaloperonospora arabidopsidis* (*Hpa*) have been shown to localize to the host cell nucleus, or to physically interact with transcriptional machinery (Mukhtar et al., 2011; Caillaud et al., 2012, 2013). Several endogenous *Arabidopsis* genes have been shown to be involved in disease susceptibility (Lapin and Van den Ackerveken, 2013; Zeilmaker et al., 2015) and expression of these may be induced by a pathogen to aid infection. Thus, being able to understand the transcriptional response to infection is not only important to understand the mechanisms by which plants resist pathogens, but also those by which pathogens suppress the plant immune system and exploit the endogenous molecular machinery of the plant for their own gain.

The pathosystem of *Arabidopsis* and its downy mildew pathogen *Hpa* has been an invaluable model in plant pathology over the past two decades for a number of reasons (Coates and Beynon, 2010). Firstly, *Hpa* is an oomycete, making it phylogenetically distinct from the many bacterial and fungal pathogens that have received extensive study, but more closely related to the agriculturally important potato blight, *Phytophthora infestans*. Additionally, the remarkable number of *Hpa* isolates, along with the number of differentially susceptible and resistant *Arabidopsis* ecotypes, available for study has made the pathosystem a useful tool for studying gene-for-gene resistance (Holub, 2007). Following this, advancements in genomics have shifted the focus toward large-scale identification of *Hpa*'s RxLR effectors and unraveling their effects on the host (Baxter et al., 2010; Fabro et al., 2011; Mukhtar et al., 2011; Caillaud et al., 2013).

Finally, the pathosystem is perhaps the clearest example of obligate biotrophy in *Arabidopsis*. Upon landing on a leaf surface, an asexual *Hpa* conidiospore germinates and forms an appressorium to penetrate the leaf surface. As early as 1 day post-infection, *Hpa* grows intercellularly as hyphae, before forming lobe-shaped structures called haustoria in almost every cell it contacts during a compatible interaction. These haustoria are invaginations of the plant cell that, while keeping the cell membrane intact, form an intimate interface between host and pathogen that aids nutrient acquisition and the delivery of effectors. Assuming successful infection, *Hpa* completes its life cycle within around 7 days, producing both asexual spores, which are carried by the tree-like conidiophores that emerge from the stomata, and sexual oospores (Coates and Beynon, 2010).

Whereas, progress is being made in identifying the key determinants of pathogenicity in *Hpa* and their effect on the host *Arabidopsis*, this progress is limited in comparison to other pathogens such as *P. syringae*, most notably because *Hpa* cannot be genetically manipulated. Several studies have looked at transcriptional change in response to *Hpa* infection (Huibers et al., 2009; Hok et al., 2011; Wang et al., 2011a; Asai et al., 2014), but it has been suggested that many of the key transcriptional events, which may occur exclusively in haustoriated cells, are often diluted by the comparative abundance of non-haustoriated cells when taking whole-organ samples (Huibers et al., 2009; Asai et al., 2014). Moreover, very little is known about the localization of *Arabidopsis* responses to *Hpa*, and how events which occur in haustoriated cells may differ from more systemic signaling events on a genome-wide scale. Making this distinction may be crucial in understanding how the haustorial environment influences the behavior of host cells.

In order to identify plant gene expression responses specifically in haustoriated cells, and to compare these to more systemic changes in gene expression during *Hpa* infection, we developed a method of isolating haustoriated cells from seedlings infected with the compatible *Hpa* isolate Noks1. The issue of dilution of highly localized pathogen responses has been previously overcome in the *Arabidopsis*-powdery mildew interaction in one published study, where by isolating infected cells through laser capture microdissection sensitivity of transcriptomic analysis was greatly increased (Chandran et al., 2010). Here, however, we chose to use Fluorescence Activated Cell Sorting (FACS) as it is a rapid way of isolating a large number of cells for gene expression analysis (Karve and Iyer-Pascuzzi, 2015). FACS is a flow cytometry technique that allows sorting of individual cells according to their fluorescence properties (Rogers et al., 2012), and has been a valuable tool for profiling the changing transcriptome of *Arabidopsis* roots during development at high spatial and temporal resolution (Brady et al., 2007). It has also been used extensively to characterize the cell type-specificity of root response to environmental/abiotic factors such as nitrogen content (Gifford et al., 2008) and salinity (Dinneny et al., 2008). FACS has also seen limited application to leaves (Grønlund et al., 2012) and analyzing the shoot apical meristem (Yadav et al., 2009), but has not been used before to study plant-pathogen interactions.

Here we used FACS to isolate haustoriated (*Hpa*-proximal) and non-haustoriated (*Hpa*-distal) cells from *Hpa* Noks1-inoculated *Arabidopsis* seedlings using the *Hpa*-responsive transgene *Pro_DMR6_:GFP* at two time points. We demonstrated that the FACS-isolated cells can be used for transcriptional analysis, and identified 278 transcripts that are differentially expressed between the cell types, relative to uninfected controls or between the two time points. Included in these transcripts were many novel responses which may give us new insight into how infection-site-specific events may influence the outcome of downy mildew infection in *Arabidopsis*.

## Materials and methods

### Plant material and growth conditions

A 2.5 kb fragment of the *DMR6* [At5g24530, *Downy Mildew Resistant 6* (van Damme et al., 2008)] promoter was PCR-amplified from *Arabidopsis* (ecotype Col-0) using the primers proDMR-F (*AAAAAGCAGGCTTCACC*GACTCTGTCTGAGTCTGAAGTCCCAAACCATG) and proDMR-R (*CAAGAAAGCTGGGT*GCCGCCATTTGATGTCAGAAAATTGAAGAAG), followed by a second amplification with pAttB1 (GGGGACAAGTTTGTACAAAAAAGCAGGCT) and pAttB2 (GGGGACCACTTTGTACAAGAAAGCTGGGT), and cloned into the pDONRZeo plasmid (Invitrogen). The entry clone was then recombined with the binary vector pBGWFS7 (Karimi et al., 2002). The resulting plasmid was then introduced into *Agrobacterium tumefaciens* strain GV3101. *Arabidopsis thaliana* Col-0 plants were transformed using the *Agrobacterium*-mediated floral dipping technique (Clough and Bent, 1998), and successful transformant seeds selected on BASTA. Homozygous T_3_ plants with single insertions were used for all experiments. *Pro_DMR6_:GFP* and Col-0 seeds were stratified for a minimum of 24 h before sowing onto soil, and were loosely covered with plastic film to retain moisture for the first 4 days after sowing. Plants were grown in a growth chamber (Weiss Technik, Vejle, Denmark) at 20°C with 10 h of light. The whole experiment was carried out in triplicate.

### *Hyaloperonospora arabidopsidis* (Hpa) propagation and inoculation

*Hpa* isolate Noks1 (Rehmany et al., 2005) was maintained on *Arabidopsis* Col-0 by weekly transfer to 7-day-old seedlings. Inoculum was collected from seedlings and sprayed at a concentration of 30,000–60,000 spores ml^−1^ onto new hosts according to Tomé et al. (2014). Spores were applied to 7-day-old seedlings carrying the *Pro_DMR6_:GFP* transgene, or Col-0 wild type. These plants were then placed in water-tight propagator trays and incubated in a growth chamber (Weiss Technik, Vejle, Denmark) at 18°C with 10 h of light.

### Imaging and microscopy

Images were acquired using a Zeiss LSM 710 confocal microscope, in conjunction with the Zeiss ZEM software.

### Protoplast generation and fluorescence activated cell sorting (FACS)

Protoplasts were generated from seedling leaves according to Grønlund et al. (2012), but with the following alterations: (i) ProtectRNA and Actinomycin D were not used, (ii) vacuum infiltration was omitted, (iii) petri dishes were rotated on orbital shaker for only 45–60 min, and (iv) only one wash and centrifugation step was performed. FACS was performed according to Grønlund et al. (2012), using a workspace derived from **Figure 3** of the publication. Cells were sorted directly into tubes containing 1 ml RLT cell lysis buffer (Qiagen) containing 1% β-mercaptoethanol, then samples stored at −80°C.

### RNA extraction, cDNA amplification, and labeling

RNA was extracted using the RNeasy Plant Mini Kit according to manufacturers instructions (Qiagen). DNase treatment was performed on-column using TURBO DNase (Life Technologies), with dose dependent on the approximate number of sorted cells in the sample, as manufacturers instructions: GFP-positive samples, which typically contained ~20,000 cells, were treated with one unit of TURBO DNase and incubated at 37°C for 20 min; all other samples, which contained >100,000 cells, were given a second equal round of DNase I treatment. cDNA was amplified using the Ovation Pico WTA System (NuGen), then labeled with Cy3 using the One-Color DNA Labeling Kit (NimbleGen) according to manufacturers instructions. RNA integrity was measured using a 2100 Bioanalyzer Picochip (Agilent). cDNA and Cy3-labeled cDNA were quantified using a NanoDrop Spectrophotometer (Thermo Scientific).

### Microarray hybridization and data normalization

Labeled cDNA samples were randomized and hybridized for 18 h on a 12x135k expression array custom designed for the TAIR10 *A. thaliana* genome annotation (Design ID OID37507; see GEO GSE58046, NimbleGen), then the arrays were washed, dried and scanned according to manufacturers instructions. The scanned microarray images were imported into DEVA software, and data outputted as raw.xys files. The data were then imported into R (R Development Core Team, 2008). The Robust Multichip Average (RMA) algorithm was used to normalize the data, taking outlier probes into account, and to summarize expression at the transcript level using median polish (Irizarry et al., 2003). All raw and normalized microarray data has been deposited in GEO (GSE67100).

### Microarray data analysis

Linear Models for Microarray Data (package *limma* in R) was used to fit linear models to pairs of samples (Figure S3), identifying genes that contrasted the most between the experimental pairs (Smyth, 2004). Transcripts were differentially expressed if they showed an absolute log_2_ fold-change of ≥0.75 [a threshold previously used by Huibers et al. (2009)] and a Benjamini-Hochberg adjusted *p* ≤ 0.05 in at least one comparison. Published data was processed in the same way, except for the data from Huibers et al. (2009), which had been previously normalized. The Cytoscape plugin BiNGO was used to identify gene ontology (GO) terms overrepresented in transcript groups, using the default settings and the “GO full” database, and a significance threshold of Benjamini-Hochberg adjusted *p* ≤ 0.05. For grouping, transcripts found to be differentially expressed in any pairwise comparison between sample types at either 5 or 7 days post-inoculation (d.p.i.) were placed in order of their ratio of proximal change to distal change, measured as log_2_(Expression_Proximal_/Expression_Control_)/log_2_(Expression_Distal_/Expression_Control_) and divided evenly into the final number of groups.

## Results

### *Pro_DMR6_*::GFP as a fluorescent reporter for host cells containing *Hyaloperonospora arabidopsidis* haustoria

In order to identify transcriptional events in *A. thaliana* that occur specifically in cells containing *Hpa* haustoria, we developed a method of using FACS to isolate haustoriated cells and non-haustoriated cells from *Hpa*-infected plants. This required a fluorescent reporter that is expressed specifically in haustoriated cells. van Damme et al. (2008) recently characterized the *Arabidopsis* gene Downy Mildew Resistant 6 (*DMR6*), which encodes a 2-oxoglutarate (2OG)-Fe(II) oxygenase and is required for susceptibility to *Hpa* isolate Waco9. By expressing a GUS reporter under the control of the *DMR6* promoter they demonstrated that *DMR6* expression is induced specifically in haustoriated cells, in both compatible and incompatible interactions with *Hpa* (van Damme et al., 2008). In order to assess *Pro_DMR6_* as a marker for isolating haustoriated cells using FACS, a construct containing 2.5 kb upstream of *DMR6* was fused to the GFP coding sequence and used to transform *Arabidopsis* Col-0 plants.

**Figure 1:**
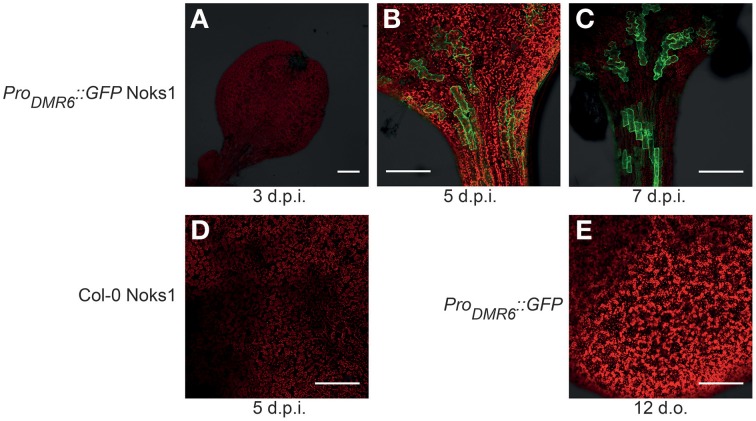
**Confocal microscopy images of** ***Hyaloperonospora arabidopsidis*** **infection marker** ***Pro_DMR6_::GFP*** **expression in *Arabidopsis* seedlings**. Scale bar represents 0.25 mm. **(A)** Absence of GFP expression in *Pro_DMR6_::GFP* seedlings infected with *Hpa* isolate Noks1, 3 d.p.i. **(B,C)** GFP expression in *Pro_DMR6_::GFP* seedlings infected with Noks1 at **(B)** 5 d.p.i. and **(C)** 7 d.p.i. **(D)** Absence of GFP expression in a Noks1-inoculated Col-0 seedling, 5 d.p.i. **(E)** Absence of GFP expression in uninfected *Pro_DMR6_::GFP* transgenic seedling; seedling is 12 days old, an equivalent age to a 5 d.p.i. seedlings.

To investigate *Pro_DMR6_::GFP* expression we screened 10-to-14-day-old T_3_ seedlings of four independent transformants using confocal microscopy. GFP expression was observed consistently in all transformants upon inoculation with the compatible *Hpa* isolate Noks1, and all transgenic lines behaved as Col-0 in terms of growth and development. Although van Damme et al. (2008) reported expression of *Pro_DMR6_::GUS* as early as 2 d.p.i., we observed little or no fluorescence at 3 d.p.i. (Figure 1A). Instead, we observed strong fluorescence at 5 (Figure 1B) and 7 d.p.i. (Figure 1C). Fluorescent cells were observed adjacent to each other, suggestive of the pattern of *Hpa* infection (Figure S1), and this was confirmed to correlate with the visibility of conidiophores on the cotyledon surface at 7 d.p.i. (data not shown). We did not observe green fluorescence in Noks1-infected Col-0 seedlings (Figure 1D), or uninoculated *Pro_DMR6_::GFP* seedlings (Figure 1E), at any time point, confirming that the GFP was expressed specifically upon *Hpa* infection in the marker line.

### Fluorescence activated cell sorting to isolate haustoriated and non-haustoriated cells from infected tissues

**Figure 2:**
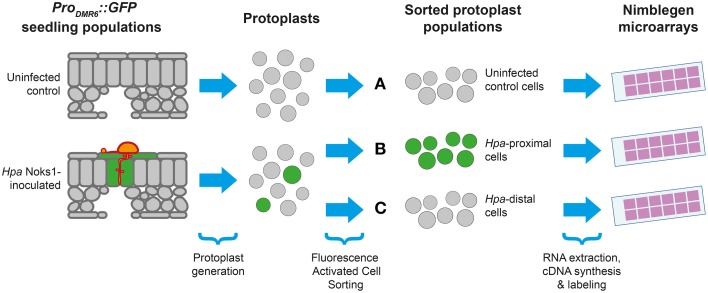
**Experimental design for studying the** ***Arabidopsis*** **response to** ***Hyaloperonospora arabidopsidis*** (***Hpa***). Protoplasts were generated from populations of seedlings containing the *Pro_DMR6_::GFP* transgene, with or without *Hpa* Noks1 infection, and sorted using Fluorescence Activated Cell Sorting to yield three types of cell population: **(A)** uninfected control cells, **(B)** GFP-positive cells from infected plants (*Hpa*-proximal cells), and **(C)** GFP-negative cells from infected plants (*Hpa*-distal cells). Whole genome expression profiling was performed from isolated cells at two time points (5 and 7 d.p.i), each with three biological replicates.

Having isolated an effective and specific marker of *Hpa* haustoriated cells, we designed an experiment allowing us to study the transcriptional response of *Arabidopsis* to *Hpa* Noks1 on a spatial scale (Figure 2). Seven-day-old *Pro_DMR6_::GFP* seedlings were inoculated with *Hpa* isolate Noks1 and cotyledons sampled at 5 and 7 d.p.i. in three biological replicates. We chose 5 d.p.i., as this was when we could first observe GFP expression under the microscope, and 7 d.p.i., as it represents a point where the *Hpa* life cycle has completed (Coates and Beynon, 2010). Protoplasts were generated from these samples and cells sorted using FACS to obtain two cell populations: GFP-expressing cells, representing the haustoriated cell population and hereon referred to as “*Hpa*-proximal cells,” and non-GFP-expressing cells, representing the non-haustoriated cell population from infected plants, hereon be referred to as “*Hpa*-distal cells.” As a control and baseline for comparison, uninfected *Pro_DMR6_::GFP* seedlings of the same age were also sampled at both time points, protoplasts generated and sorted through FACS.

Protoplasts were generated using a recent protocol for FACS of leaf cells by Grønlund et al. (2012). Immediately prior to FACS, a small subset of the protoplasts derived from infected seedlings express GFP, consistent with the proportion of GFP expressing cells in infected seedling leaves. This GFP expression was detected upon FACS analysis (Figure S2). In contrast, GFP expressing cells were not observed in protoplasts from uninfected seedlings prior to FACS. From the 18 protoplast samples collected (three cell populations × two time points × three biological replicates), RNA was extracted, converted to cDNA, labeled, and hybridized to whole genome oligonucleotide *Arabidopsis* microarrays.

### Differential expression of genes in *Hpa*-proximal and *Hpa*-distal cells gives insight local and systemic responses to the pathogen

Microarray gene expression was summarized at the transcript level and normalized using the RMA algorithm (Irizarry et al., 2003) (Table S1). In order to identify transcripts which were differentially expressed (DE) in *Hpa*-proximal cells, and to differentiate these from systemic signaling observed in cells distal to the infection site, we performed pairwise comparisons (Figure S3) across cell populations and time points using Linear Models for Microarray Data (LIMMA) (Smyth, 2004). A total of 278 transcripts were identified as differentially expressed at a cutoff of absolute log_2_ fold-change ≥0.75 and a Benjamini-Hochberg adjusted *p*-value ≤ 0.05 in at least one pairwise comparison (Table S2).

As a confirmation that the cells isolated by FACS were those that were *Hpa*-associated, among the 278 DE transcripts was *DMR6* (At5g24530), which showed ~seven-fold upregulation in *Hpa*-proximal cells relative to uninfected control cells at 7 d.p.i. (Benjamini-Hochberg adjusted *p* = 0.035), and at 5 d.p.i. (Benjamini-Hochberg adjusted *p* = 0.061). We also observed upregulation of several other genes which have been previously implicated in the *Hpa* response, or as more general regulators of plant-pathogen interactions. These include Impaired Oomycete Susceptibility 1 (*IOS1*, At1g51800), Pathogenesis-Related 4 (*PR4*, At3g04720), Pathogen and Circadian Controlled 1 (*PCC1*, At3g22231), Flg22-induced Receptor-like Kinase 1 (*FLK1*, At2g19190) and *WRKY8* (At5g46350) (Table S2).

**Figure 3:**
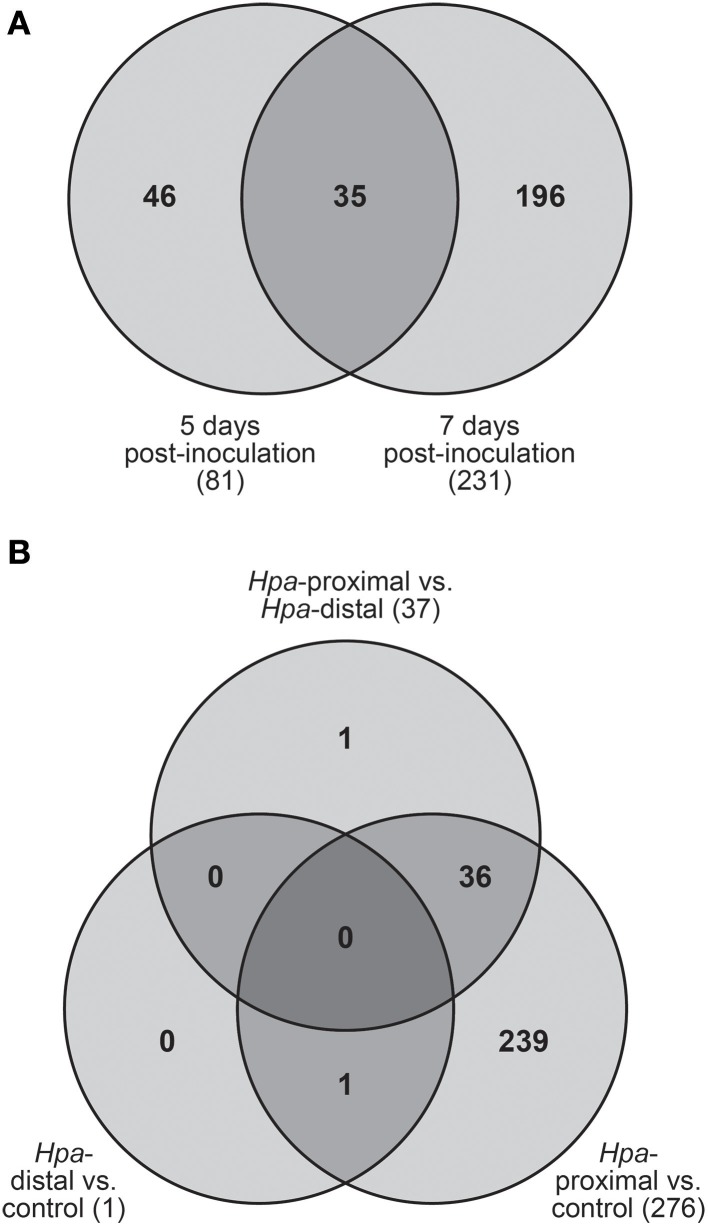
**Transcripts found to be differentially expressed (DE) using pairwise contrasts in LIMMA. (A)** Distribution of DE transcripts across the two time points. The vast majority of transcripts were found to be DE at 7 d.p.i. **(B)** Number of DE transcripts identified when making pairwise contrasts between cell types, taking into account both 5 and 7 d.p.i. The vast majority of transcripts were DE between *Hpa*-proximal cells and control cells from uninfected plants.

Of the 278 total DE transcripts, 81 and 231 transcripts were DE between the three cell types at 5 d.p.i. and 7 d.p.i. respectively, with 35 transcripts being DE over both time points (Figure 3A). 276 transcripts were DE between *Hpa*-proximal cells and uninfected control cells from the same time point, with 37 transcripts found to be DE between *Hpa*-proximal and *Hpa*-distal cells at the same time point (Figure 3B). A single transcript, At2g18660.1 (Plant Natriuretic Peptide A, PNP-A), was found to be DE between GFP-negative (*Hpa*-distal) cells from infected plants and cells from uninfected plants. Together with the detection of previously characterized *Hpa* responsive genes, the observation that the vast majority of transcriptional responses are being identified in the *Hpa*-proximal populations, rather than the *Hpa*-distal populations, from infected plants confirms that *Hpa*-responsive cells can be isolated using FACS.

In order to discover what types of genes are responding locally vs. systemically, i.e., specifically in *Hpa*-proximal cells vs. more generally in both *Hpa*-proximal and *Hpa*-distal cells, the 278 DE transcripts were grouped according to the localization of their response at each of the time points, and these groups were searched for overrepresentation of GO terms using the Cytoscape plugin BiNGO (Benjamini-Hochberg adjusted *p* ≤ 0.05, Maere et al., 2005, Figure 4, Table S3). To take a more granular view of response location we chose to differentiate local and systemic genes based on the ratio of their *Hpa*-proximal response (log_2_ fold-change relative to uninfected control) to their *Hpa*-distal response; for a list of the genes within each group, see Table 1, Table S2.

**Figure 4:**
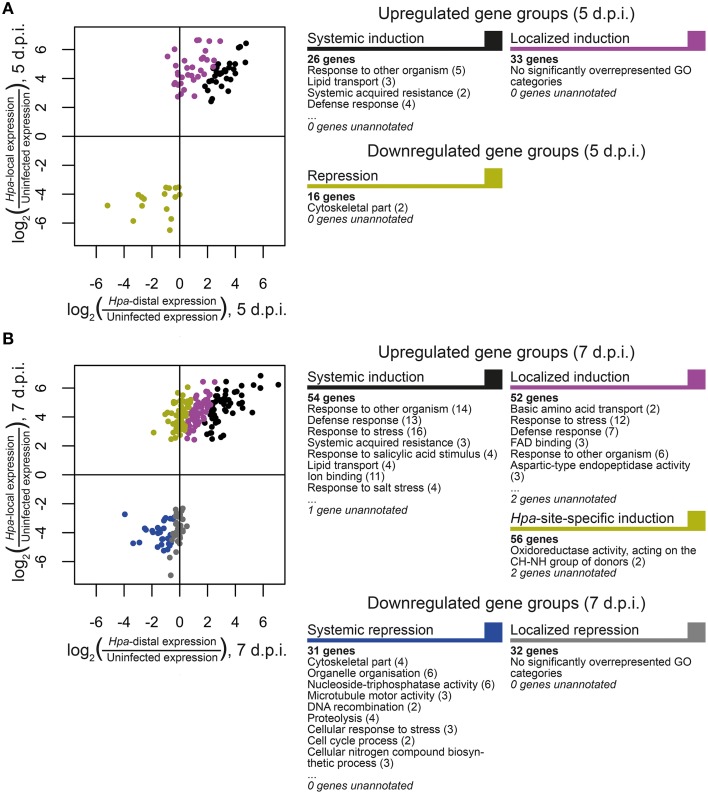
**Grouping of differentially expressed** ***Arabidopsis*** **genes according to the direction and localization of response**. Genes were grouped at **(A)** 5 d.p.i. and **(B)** 7 d.p.i. according to the nature of their response at that time point. Scatterplots display the ratio of *Hpa*-local and *Hpa*-distal expression to expression in uninfected control cells for each gene in each group. Selected GO categories that are overrepresented (Benjamini-Hochberg adjusted *p* ≤ 0.05) in each gene group are also displayed, with the number of associated genes in parentheses. A full list of overrepresented GO terms, their number IDs, with cluster and genomic frequencies and Benjamini-Hochberg adjusted *p*-values can be seen in Table S3.

**Table 1:** **Differentially expressed genes grouped according to the direction and localization of their response at 5 and 7 d.p.i**.

**Groups at 5 d.p.i.**							
**SYSTEMIC INDUCTION (26 GENES)**				**LOCALIZED INDUCTION (33 GENES)**			
**ID**	**Name**	**ID**	**Name**	**ID**	**Name**	**ID**	**Name**
AT1G02850	BGLU11	AT3G49210		AT1G06770	DRIP1	AT3G09940	ATMDAR3,
AT1G14880	PCR1	AT3G49620	DIN11	AT1G25390		AT3G11340	
AT1G35710		AT3G53600		AT1G27020		AT3G14225	GLIP4
AT1G49050		AT4G11890		AT1G30730		AT3G22600	
AT1G73805		AT4G12480	pEARLI 1	AT1G35260	MLP165	AT3G25655	IDL1
AT1G74710	EDS16, ATICS1	AT4G12500		AT1G44130		AT3G60120	BGLU27
AT2G25510		AT4G20000		AT1G51800		AT4G11890	
AT2G27660		AT4G21850	MSRB9	AT1G53470	MSL4	AT4G23550	WRKY29
AT3G04720	PR4, HEL	AT4G23150	CRK7	AT1G61120	TPS04, GES	AT4G25950	VATG3
AT3G22231	PCC1	AT5G03350		AT1G69930	GSTU11	AT4G38830	CRK26
AT3G22235		AT5G13320	ATGH3.12	AT1G74010		AT5G12340	
AT3G25610		AT5G40780		AT2G19500	CKX2, ATCKX2	AT5G22540	
AT3G29034		AT5G44568		AT2G20805		AT5G37490	
**REPRESSION (16 GENES)**				AT2G28110	FRA8, IRX7	AT5G38900	
**ID**	**Name**	**ID**	**Name**	AT2G31990		AT5G39580	
AT1G05910		AT3G28460		AT2G36810		AT5G48657	
AT1G71050	HIPP20	AT3G52770	ZPR3	AT3G02240	RGF7		
AT1G71695		AT4G00400	GPAT8,				
AT1G73620		AT5G02770					
AT2G22400		AT5G13000	gsl12				
AT2G32860	BGLU33	AT5G17410					
AT3G18160	PEX3-1	AT5G40640					
AT3G19960	ATM1	AT5G58240	FHIT				
**Groups at 7 d.p.i.**							
**SYSTEMIC INDUCTION (54 GENES)**				**LOCALIZED INDUCTION (52 GENES)**			
**ID**	**Name**	**ID**	**Name**	**ID**	**Name**	**ID**	**Name**
AT1G01680	PUB54	AT3G29130		AT1G02850	BGLU11	AT3G21710	
AT1G02520	PGP11	AT3G47480		AT1G05260	RCI3, RCI3A	AT3G46616	
AT1G02850	BGLU11	AT3G61280		AT1G26420		AT3G52710	
AT1G14880	PCR1	AT4G01350		AT1G30720		AT3G61390	
AT1G19610	LCR78, PDF1.4	AT4G12480	pEARLI 1	AT1G30730		AT4G01720	WRKY47
AT1G33960	AIG1	AT4G12490		AT1G34460	CYCB1;5, CYC3	AT4G09420	
AT1G35710		AT4G12500		AT1G34670	MYB93	AT4G16563	
AT1G55790		AT4G14400	ACD6	AT1G44130		AT4G18540	
AT1G66280	BGLU22	AT4G16260		AT1G53830	PME2	AT4G21120	AAT1, CAT1
AT1G73805		AT4G20000		AT1G56550	RXGT1	AT4G23210	CRK13
AT1G74710	EDS16, ICS1, SID2	AT4G20110	VSR7, VSR3;1, BP80-3;1	AT1G58190	RLP9	AT4G38830	CRK26
AT1G75040	PR5, PR-5	AT4G23150	CRK7	AT1G58400		AT5G02230	
AT2G14560	LURP1	AT4G39830		AT1G64583		AT5G15130	WRKY72
AT2G18660	PNP-A	AT5G01900	WRKY62	AT1G65090		AT5G18780	
AT2G21900	WRKY59, ATWRKY59	AT5G10760		AT1G66920		AT5G21280	
AT2G44380		AT5G11210	GLR2.5	AT1G69930	GSTU11	AT5G22570	WRKY38
AT2G44890	CYP704A1	AT5G13320	PBS3, GDG1, WIN3, GH3.12	AT1G71910		AT5G25260	
AT2G45510	CYP704A2	AT5G22540		AT1G77380	AAP3	AT5G25910	RLP52
AT3G04720	PR4, HEL	AT5G24530	DMR6	AT2G19190	FRK1	AT5G37415	AGL105
AT3G09940	MDHAR, MDAR3,	AT5G37490		AT2G27180		AT5G37540	
AT3G11340		AT5G37600	GLN1;1, GSR 1	AT2G28110	FRA8, IRX7	AT5G38540	
AT3G18250		AT5G38550		AT2G30550		AT5G48290	
AT3G21080		AT5G38900		AT2G35980	YLS9, NHL10	AT5G50200	WR3, NRT3.1
AT3G22235		AT5G39580		AT2G45220		AT5G57450	XRCC3
AT3G22600		AT5G44585		AT2G47550		AT5G59930	
AT3G26210	CYP71B23	AT5G44920		AT3G09940	MDHAR MDAR3, MDAR2	AT5G61640	PMSR1, ATMSRA1
AT3G29034		AT5G46350	WRKY8				
***Hpa*-SITE-SPECIFIC INDUCTION (56 GENES)**							
**ID**	**Name**	**ID**	**Name**	**ID**	**Name**	**ID**	**Name**
AT1G01150		AT1G76370		AT3G50190		AT4G37710	
AT1G05880	ARI12	AT2G09840		AT3G54730		AT5G06520	
AT1G13480		AT2G19500	CKX2	AT3G55150	EXO70H1	AT5G07610	
AT1G15640		AT2G21550		AT3G55700		AT5G11400	
AT1G17020	SRG1	AT2G30395	OFP17	AT3G60120	BGLU27	AT5G18270	ANAC087
AT1G21360	GLTP2	AT2G35770	scpl28	AT3G61827		AT5G19270	
AT1G29600		AT2G38365		AT3G62640		AT5G20330	BETAG4
AT1G51915		AT2G43730		AT4G01750	RGXT2	AT5G24080	
AT1G53980		AT3G01420	ALPHA-DOX1	AT4G03950		AT5G28190	
AT1G60095		AT3G06260	GATL4	AT4G04775		AT5G28235	
AT1G61750		AT3G14225	GLIP4	AT4G14630	GLP9	AT5G39560	
AT1G63245	CLE14	AT3G15340	PPI2	AT4G15417	RTL1	AT5G42120	
AT1G68630		AT3G26470		AT4G19950		AT5G61160	AACT1
AT1G69810	WRKY36	AT3G29035	ANAC059, NAC3	AT4G20470		AT5G63225	
**SYSTEMIC REPRESSION (31 GENES)**				**LOCALIZED REPRESSION (32 GENES)**			
**ID**	**Name**	**ID**	**Name**	**ID**	**Name**	**ID**	**Name**
AT1G10930	ATSGS1, RECQ4A	AT3G04460	PEX12, APM4	AT1G07320	RPL4	AT2G36490	DML1, ROS1
AT1G12244		AT3G04850		AT1G11720	ATSS3, SS3	AT3G05730	
AT1G16350		AT3G23670	PAKRP1L, KINESIN-12B	AT1G12845		AT3G15353	MT3
AT1G21440		AT3G28460		AT1G13380		AT3G19450	ATCAD4,
AT1G48620	HON5	AT4G11990		AT1G14690	MAP65-7	AT3G45850	
AT1G48650		AT4G21270	ATK1, KATAP	AT1G27385		AT3G54190	
AT1G54820		AT4G22930	PYR4, DHOASE	AT1G35780		AT3G60840	MAP65-4
AT1G58060		AT4G30610	BRS1, SCPL24	AT1G48600	AtPMEAMT	AT4G37080	
AT1G63630		AT4G34210	ASK11, SK11	AT1G53560		AT5G01015	
AT1G66510		AT4G37110		AT1G70370	PG2	AT5G15310	ATMYB16, ATMIXTA,
AT2G21380		AT5G17220	GST26, TT19, GSTF12	AT1G71695		AT5G15740	
AT2G22330	CYP79B3	AT5G17410		AT1G79200		AT5G20630	ATGER3,
AT2G26680		AT5G46390		AT1G79280	NUA, AtTPR	AT5G39790	
AT2G40760		AT5G51350		AT2G26330	ER, QRP1	AT5G48600	ATCAP-C
AT2G45440	DHDPS2	AT5G64240	MC3	AT2G30540		AT5G50740	
AT3G02900					AT2G32880		AT5G57130

At 5 and 7 d.p.i., transcripts were classified as either “upregulated” or “downregulated,” then split into groups according to the ratio of a Hpa-local response (measured as the log_2_ fold-change between Hpa-local cells and uninfected cells at that time point) and a Hpa-distal response (the fold-change between Hpa-distal and uninfected cells). See Figure 4 for a graphical representation of their expression pattern, Table S2 for expression values, and Table S3 for GO term analysis details.

The 81 transcripts DE at 5 d.p.i. were split into three groups (Figure 4A). For upregulated genes, we were interested in broadly comparing local and systemic responses, so we split the transcripts found to be upregulated at this time point into two groups—one representing systemic induction (almost equal proximal and distal response), and one representing localized induction (strong proximal response, weak distal response). The systemic induction group showed overrepresentation of pathology-related GO terms such as “response to other organism” and “defense response,” as well as “systemic acquired resistance,” fitting to the systemic expression pattern of the genes in this group. This suggests that, despite the lack of genes DE in *Hpa*-distal cells relative to the control, this population of cells is capturing systemic signaling in response to *Hpa*. Genes involved in lipid transport and localization were also overrepresented in this group. Individual genes represented in this group include Enhanced Disease Susceptibility to *Erysiphe orontii* (*EDS16*, At1g74710) and AVRPPHB Susceptible 3 (*PBS3*, At5g13320), which have both been implicated in salicylic acid accumulation in plant defense (Wildermuth et al., 2001; Nobuta et al., 2007), and Lysine Histidine Transporter 1 (*LHT1*, At5g40780), which has been shown to influence plant defense in a salicylic acid-mediated manner (Liu et al., 2010). The defense genes Pathogenesis-Related 4 (*PR4*, At3g04720) and Pathogen and Circadian Controlled 1 (*PCC1*, At3g22231) also fell into this group.

In contrast localized induction group did not show overrepresentation of any GO terms, suggesting a diversity of genes within this group. Individual genes represented in this group include the transcription factor *WRKY29* (At4g23550), a terpene synthase (*TPS4*, At1g61120) and a peroxidase superfamily protein (At5g39580). The group also includes cysteine-rich receptor-like protein kinases *ARCK1* (At1g11890) and *CRK26* (At4g38830), and a monodehydroascorbate reductase (*AtMDAR3*, At3g09940) that is crucial for colonization of *Arabidopsis* by the mutualistic fungus *Piriformospora indica* (Vadassery et al., 2009).

Due to the small number of transcripts at this time point, downregulated genes could not effectively be split into “systemic” and “local” responding and were thus considered as one group. This group showed overrepresentation for only one GO term: “cytoskeletal part.” Downregulated genes include Callose Synthase 3 (At5g13000), peroxidase 12 (At1g71695) and a pathogenesis-related thaumatin superfamily protein (At1g73620).

The larger number (231) of transcripts DE at 7 d.p.i. allowed us to split them into more groups (Figure 4B). Transcripts upregulated at this time point were this time split into three gene groups—systemic induction, local induction and infection-site-specific induction, representing increasing localization of their response, such that genes in the infection-site-specific induction group showed a negligible *Hpa*-distal response. As with the systemic induction group at 5 d.p.i., the systemic induction group at 7 d.p.i. showed overrepresentation for the GO terms “lipid transport,” “systemic acquired resistance” and a number of generic defense-related terms such as “defense response.” The GO terms “response to salicylic acid stimulus” and “response to stress” were also additionally overrepresented in this group. Individual genes within this group include Pathogenesis-Related 4 (*PR4*, At3g04720) and 5 (*PR5*, At1g75040), *WRKY59* (At2g21900), *WRKY62* (At5g01900) and *WRKY8* (At5g46350), Accelerated Cell Death 6 (*ACD6*, At4g14400), Plant Natriuretic Peptide A (*PNP-A*, At2g18660) and Late Upregulated in Response to *Hyaloperonospora parasitica* (*LURP1*, At2g14560). Surprisingly, *DMR6* fell into this group, despite being used as our marker for *Hpa*-local cells. This could be due to weaker, more systemic signaling of *DMR6* that was beyond detection using a GFP marker. As this data set is enriched for responses predominantly in *Hpa*-local cells, this too may also been an indication that even the most systemic responses captured remain fairly localized to the infection site.

The local induction group showed similar GO term enrichment to the systemic induction group at 7 d.p.i., such as the pathology-related terms “response to other organism” and “defense response” and the more generic “response to stress.” A number of receptor-like proteins were present in this group, including Flg22-induced Receptor-like Kinase 1 (*FRK1*, At2g19190), Cysteine-rich Receptor-like Kinase 13 (*CRK13*, At4g23210), Receptor Like Proteins 9 (*AtRLP9*, At1g58190) and 52 (*AtRLP52*, At5g25910) a putative CC-NBS-LRR class disease resistance protein (At1g58400) and a putative TIR-NBS class disease resistance protein (At4g09420). *WRKY47*, (At4g01720), *WRKY72* (At5g15130) and *WRKY38* (At5g22570) were also in this group.

Infection-site-specific induced, representing the most localized genes upregulated at 7 d.p.i., showed overrepresentation of only the GO term “oxidoreductase activity, acting on the CH-NH group of donors.” Genes in this group include the transcription factors *WRKY36* (At1g69810), *NAC3* (At3g29035) and *NAC087* (At5g18270), as well as an RNA-binding Suppressor-of-White-APricot (SWAP) protein (At5g06520).

The larger number of downregulated genes at 7 d.p.i., relative to 5 d.p.i., allowed us to split them into two groups representing systemic and local repression. Genes that showed systemic repression were overrepresented for a number of cellular functions such as “cytoskeletal part,” “organelle organization,” “cell cycle process,” and “nucleoside-triphosphatase activity.” Genes in this group include a histone H1/H5 family member (At1g48620), metacaspase 3 (*MC3*, At5g64240) and *A. thaliana* Kinesins 1 (*ATK1*, At4g21270) and 12B (*ATK12B*, At3g23670).

Finally, there was no overrepresentation of GO terms in the localized repression group. Genes in this group included peroxidase 12 (*PER12*, At1g71695), the receptor protein kinase *ERECTA* (At2g26330), microtubule-associated protein 65-4 (*MAP65-4*, At3g60840) and Starch Synthase 3 (*ATSS3*, At1g11720).

### Comparison with published data sets

To ask if the FACS approach identifies novel genes in the *Arabidopsis* response to *Hpa* infection, we compared our list of differentially expressed genes to previously published microarray data from Huibers et al. (2009), Wang et al. (2011a) and Hok et al. (2011). Data from these publications was retrieved from the relevant public databases and processed in a similar manner to the data we present here, i.e., differentially expressed genes identified by making pairwise contrasts in LIMMA. From each published dataset we considered only samples and direct comparisons that were most relevant to our experimental design here. Huibers et al. (2009) used two-color CATMA arrays to profile expression in a compatible *Arabidopsis-Hpa* interaction (Landsberg erecta (Ler) and Cala2) and an incompatible interaction (Ler and Waco9), relative to uninfected controls, at 3 d.p.i. Wang et al. (2011a) performed a 6-day timecourse of infection with the incompatible strain Emwa1, in Col-0 and the susceptible mutant *rpp4*. Finally, Hok et al. (2011) measured gene expression in *Arabidopsis* Wassilewskija (WS) seedlings after mock treatment, and treatment with the compatible isolate Emwa, at an early time point (8 and 24 h post-inoculation) and at a late time point (4 and 6 d.p.i.). For the former two datasets, we considered only the Cala2 interaction and the *rpp4* interaction, respectively, as they represented compatible interactions that result in a similar outcome to the Col-0 and Noks1 interaction, i.e., completion of the *Hpa* lifecycle. For the latter two datasets, which have multiple time points, we considered all time points as to capture as much of the *Hpa* response as possible.

**Figure 5:**
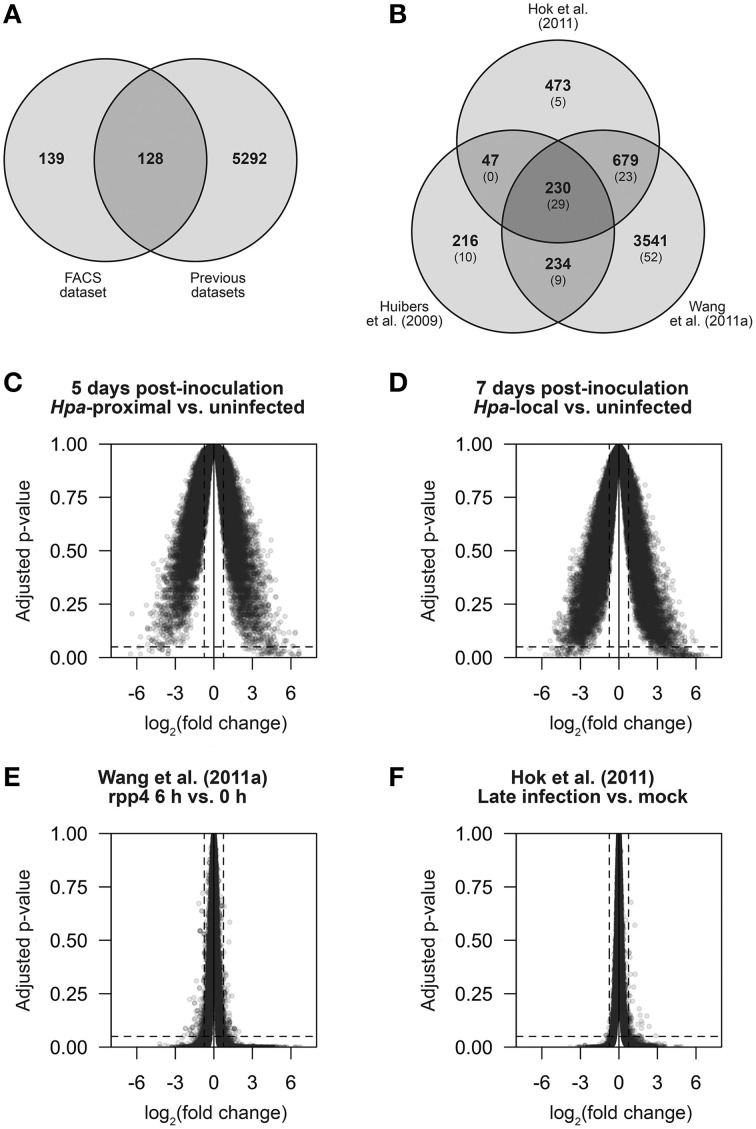
**The strength and specificity of** ***Hpa*****-responsive gene detection in multiple datasets**. In order to make direct comparisons, published data from Huibers et al. (2009), Wang et al. (2011a) and Hok et al. (2011) was processed in a similar manner to the data we present here. **(A,B)** Comparison of genes found to be differentially expressed (DE) using LIMMA. **(A)** Gene overlap of genes DE in our FACS dataset and genes found to be DE based on the previously published data. Of the 267 DE genes identified in our FACS dataset, 128 were previously detectable and 139 were novel. **(B)** Gene overlap between the three previously published datasets. In parentheses is the number of genes in each group that also overlap with our FACS dataset. **(C–F)** Average log_2_ fold change against Benjamini-Hochberg adjusted *p*-values for all measured transcripts (determined by LIMMA) in the comparisons: **(C)** *Hpa*-proximal vs. uninfected cells, 5 d.p.i. **(D)** *Hpa*-proximal vs. uninfected cells, 7 d.p.i. **(E)** Wang et al. (2011a) *rpp4* Emwa1 6 d.p.i. vs. 0 d.p.i., **(F)** Hok et al. (2011) Emwa vs. Mock (late infection). Dotted lines represent the differential expression significance thresholds of absolute log_2_ fold change ≥0.75 and adjusted *p* ≤ 0.05.

Our 278 differentially expressed transcripts represent 267 different genes—128 of which could be detected in the previously published datasets based on our analysis (Figure 5A). The remaining 139 genes are thus novel *Hpa* responses identified by our FACS-based cell response type specific approach. However, ~5300 transcripts were previously detected as differentially expressed in one or more of the datasets outlined above, but not differentially expressed in our dataset. A comparison between previous datasets shows that only a small proportion of these are common between datasets (Figure 5B), suggesting that these DE genes arose as differences in experimental design, *Hpa* strain used or otherwise may be false positives.

In order to compare the sensitivity and specificity of our approach to the previously published data, we compared the average fold-change and Benjamini-Hochberg adjusted *p*-values for all genes for a number of pairwise comparisons across different datasets (Figures 5C–F). We found that our dataset had a larger proportion of genes with significant (≥0.75) log_2_ fold changes relative to an uninfected control than in the previously published datasets, and those identified as DE showed DE of a higher magnitude, highlighting that by specifically analyzing *Hpa*-proximal cells, we observe greater sensitivity in expression changes during infection (compare the width of plots in Figures 5C,D to Figures 5E,F). Conversely, the published datasets had a larger proportion of genes within the significance threshold of adjusted *p* ≤ 0.05, but with almost-zero fold-changes (Figures 5E,F). This suggests that, relative to the published datasets, although our data shows higher sensitivity, in this instance noise may be a limiting factor in *Hpa*-responsive gene detection.

## Discussion

Here we present the novel use of FACS to isolate *A. thaliana* cells infected by the downy mildew pathogen *Hpa*. To our knowledge, this is the first use of FACS to specifically isolate plant cells responding to infection, although this has previously been achieved in animal systems (Richman et al., 2002; Thöne et al., 2007).

We demonstrate that cells isolated by FACS of *Hpa*-infected seedlings can be used for transcriptomic analysis of the local vs. systemic response to *Hpa* infection. Consistent with expectations that the majority of transcriptional events would occur at the infection site, all differentially expressed genes were either significantly upregulated or downregulated in the *Hpa*-proximal cell population, over time, or relative to an uninfected control or *Hpa*-distal cells from infection plants at the same time point. In contrast, only a single transcript showed significant differential expression between *Hpa*-distal cells and uninfected control cells. The identity of this transcript as Plant Natriuretic Peptide A (*PNP-A*, At2g18660) is assuring as PNP-A has been previously described as a secreted signal working systemically during both abiotic and biotic stress (Wang et al., 2011b). Ideally we would have identified further genes to be significantly differentially expressed in the *Hpa*-distal population, representing systemic signaling. However, as the *Hpa*-distal cell population was simply a collection of cells not expressing the haustoriated cell marker *Pro_DMR6_::GFP*, we might expect this population to be heterogeneous, containing cells at varying proximity to the pathogen, many of which may not be responding to the pathogen at all. To address this potential dilution of systemic responses, we considered that many of the genes differentially expressed in *Hpa*-proximal cells may also be responding more systemically, and grouped these into the “Systemic Induction” and “Systemic Repression” groups in Figure 4. Several of the genes and GO terms associated with these groups are consistent with what is already known about defense signaling in *Arabidopsis*, such as the role of salicylic acid and salicylic acid-responsive gene expression in systemic acquired resistance (Durrant and Dong, 2004). However, no firm conclusions can currently be made from the analysis in Figure 4 and further experiments are needed to validate the localization of these responses, and to unravel their significance in the *Hpa-Arabidopsis* interaction.

The use of FACS to study cells specifically at the site of infection has potential to increase the sensitivity of transcriptomic or other high-throughput analyses, such as proteomics. We have shown that in general, the magnitude of up- or down-regulation of genes is greater in our FACS-isolated *Hpa*-proximal cells than in previous whole-leaf datasets, relative to uninfected controls (Figures 5C–F). We have also identified a number of genes that are differentially expressed in *Hpa*-proximal cells not previously detected in microarray studies (Figure 5A). However, we have also failed to detected many genes previously associated with *Hpa* infection. While many of these could potentially be attributed to differences in experimental design or the *Hpa* isolate used, it seems that noise is largely a contributing factor. Greater optimization of the FACS protocol will hopefully help to overcome this in the future.

A crucial development in the use of FACS for studying local vs. systemic signaling during *Arabidopsis* infection will be the development of new cell markers. A key challenge, particularly for the *Hpa* pathosystem, is that the pathogen and the proteins that it delivers into host cells cannot currently be fluorescently labeled through genetic manipulation. As such, isolation of *Hpa*-contacting cells relies entirely on pathogen-responsive *Arabidopsis* promoters, which may not be induced immediately and are likely to show changes in expression over the course of infection. This seems to be an issue with the *DMR6* promoter, from which we could not detect GFP expression until 5 d.p.i. This prevented us from studying earlier stages of infection, which is unfortunate as it is at these stages that the use of FACS will be most informative, as the limited spread of the pathogen precludes the use of whole tissue microarrays. An additional caveat more relevant to this dataset, is that, at the later time points (e.g., 5 and 7 d.p.i.), recently haustoriated cells may not fluoresce, and may instead be interpreted as *Hpa*-distal cells. Characterizations of new, early-induced haustoriated cell markers, as well as an in-depth study of their expression patterns will be crucial in developing a refined FACS approach. Furthermore, to avoid dilution of the systemic response, one could use a second fluorophore to mark cells within a certain range of the pathogen. This could potentially be complex as signals and responses spread over space. In addition to developing new methods to study pathogen signaling at a cell-specific resolution, we must in turn develop theoretical methods to understand the data being generated, and perhaps take into account some of the assumptions and limitations of the FACS approach. As these methods develop, we can better understand the events that occur specifically at the *Arabidopsis-Hpa* interface, and how these might influence more widespread signaling in the plant.

## Author contributions

TC, MG, and JB contributed to design of the experiment and interpretation of the data. VC generated the *Pro_DMR6_::GFP* construct and plant lines, and TC and VC performed microscopy of these plants. Protoplast generation, FACS and microarray analysis was performed by TC. All authors wrote the manuscript.

### Conflict of interest statement

The authors declare that the research was conducted in the absence of any commercial or financial relationships that could be construed as a potential conflict of interest.

## Supplementary material

Figure S1 **Confocal microscopy images of *Hyaloperonospora arabidopsidis* (*Hpa*) infection marker *Pro_DMR6_::GFP* expression in an *Arabidopsis* cotyledon, 7 d.p.i. with compatible *Hpa* isolate Noks1**. **(A,B)** Expression of the marker follows the pattern of pathogen spread across the cotyledon. **(C–E)** Cells expressing the marker appear to contain haustoria.

Figure S2 **Fluorescence expression profiles for cells analyzed and sorted with FACS**. **(A,B)** Dot plots of output from the 580/30 nm vs. 530/40 nm bandpass filters on the BD Influx, using a workspace derived from Grønlund et al. (2012). **(A)** Protoplasts generated from uninfected, 14-day-old *Pro_DMR6_::GFP* seedlings, where cells were collected exclusively from the low 580/low 530 (GFP-negative) fate. **(B)** Protoplasts generated from *Pro_DMR6_::GFP* inoculated with *Hpa* isolate Noks1, at 7 d.p.i., where cells were collected from both the low 580/low 530 (GFP-negative) and low 580/high 530 (GFP-positive, ~0.5%) gates. The high 580/low 530 gate represents cell debris.

Figure S3 **Pair-wise comparisons used to identify differentially expressed genes**. Expression at 7 d.p.i. and 5 d.p.i. was compared for each cell type. Genes significantly differentially expressed in either *Hpa*-distal or *Hpa*-proximal cells, but not uninfected control cells, over time were considered as differentially expressed. To investigate differential expression between cell types, six comparisons were performed: *Hpa*-distal cells vs. uninfected control cells, *Hpa*-proximal cells vs. uninfected control cells, and *Hpa*-proximal cells vs. *Hpa*-distal cells, independently for each time point.

Table S1 **Normalized log_2_ expression data for uninfected control, *Hpa*-proximal, and *Hpa*-distal cells at 5 and 7 d.p.i**. Gene IDs, names, and descriptions are given based on the TAIR10 genome annotation. Column names are given in the format “Cell type, time point, replicate,” where “Control5a” is control cells at 5 d.p.i., replicate 1.

Table S2 **Transcripts differentially expressed between uninfected control, *Hpa*-proximal, and *Hpa*-distal cells at 5 and 7 d.p.i**. Gene IDs, names and descriptions are given based on the TAIR10 genome annotation. Mean log_2_ expression values are given in columns D-I–the names of these columns are given in the format “Cell type, time point,” where “Control5” is control cells at 5 d.p.i. Columns J-M are fold-changes for distal and proximal cells at 5 d.p.i. and 7 d.p.i., relative to uninfected controls at the respective time points. Columns N (“Group@5dpi”) and O (“Group@7dpi”) give the names of the groups which that transcript belonged to (see Figure 4), or state “Not DE” if not differentially expressed at that time point. Column P (“Newly detected?”) states “Yes” or “No” as to whether the gene is newly detected in our dataset, based on the analysis in Figure 5A.

Table S3 **Overrepresentation of Gene Ontology (GO) terms in *Hpa*-responsive gene groups**. *Hpa*-responsive genes were grouped according to the localization of their response at 5 and 7 d.p.i. (see Table S2, Figure 4). All GO terms found to be overrepresented (Benjamini-Hochberg adjusted *p* ≤ 0.05) in the groups are included, other than those that were only represented by a single gene in a group, which were excluded. The percentage frequency of each GO term in the cluster and in the *Arabidopsis thaliana* genome (of the 27,594 GO-annotated genes), as well as a list of genes annotated with the GO term are included.
