# Supplementary figures and images for: Spatial dissection of the Arabidopsis thaliana transcriptional response to downy mildew using Fluorescence Activated Cell Sorting
Source: Front Plant Sci. 2015 Jul 10;6:527. doi: 10.3389/fpls.2015.00527 (PMC4498041; doi:10.3389/fpls.2015.00527)

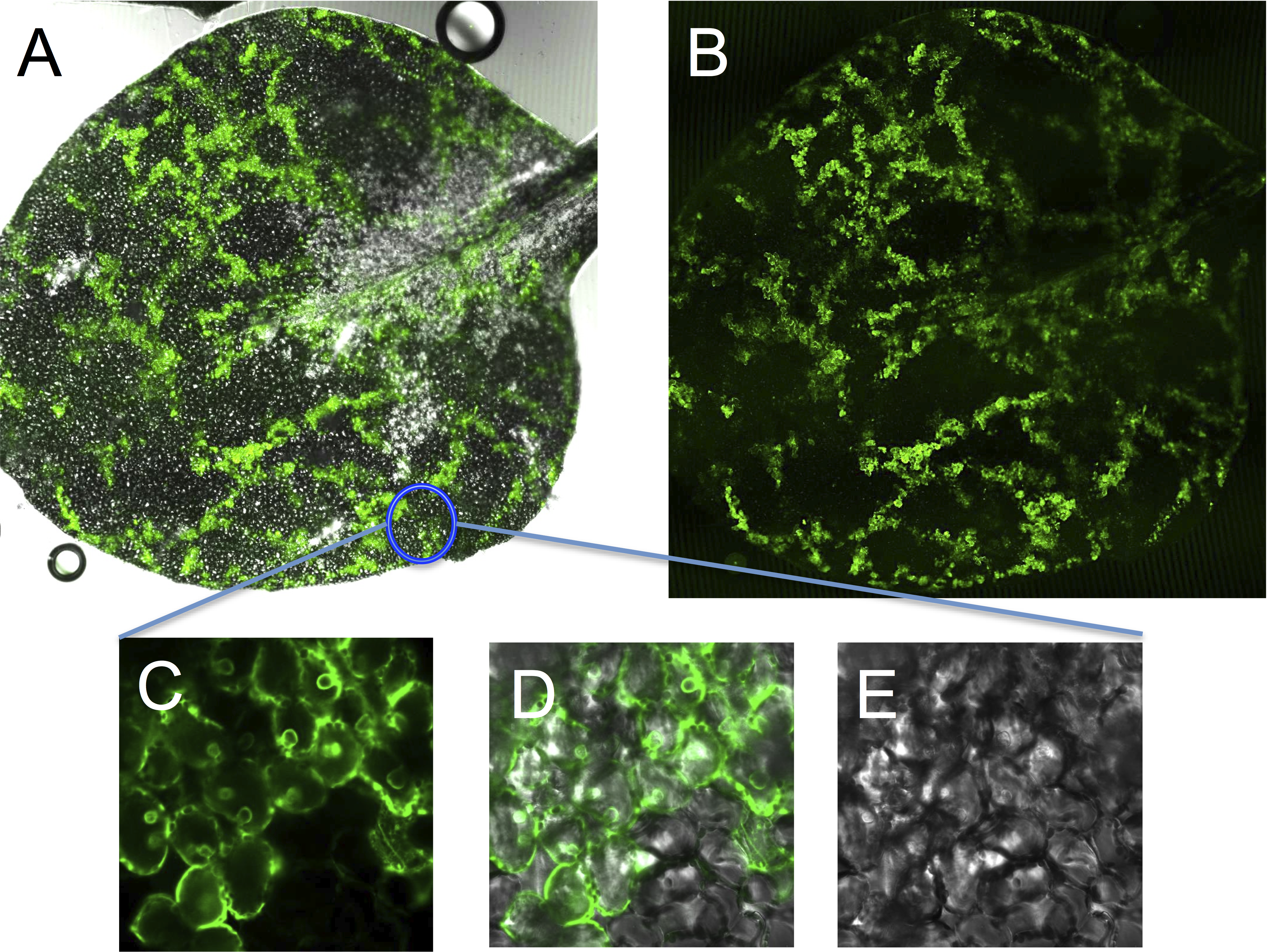

Supplement: Figure S1 — Confocal microscopy images of Hyaloperonospora arabidopsidis (Hpa) infection marker ProDMR6::GFP expression in an Arabidopsis cotyledon, 7 d.p.i. with compatible Hpa isolate Noks1. (A,B) Expression of the marker follows the pattern of pathogen spread across the cotyledon. (C–E) Cells expressing the marker appear to contain haustoria. [file FigureS1.TIF]

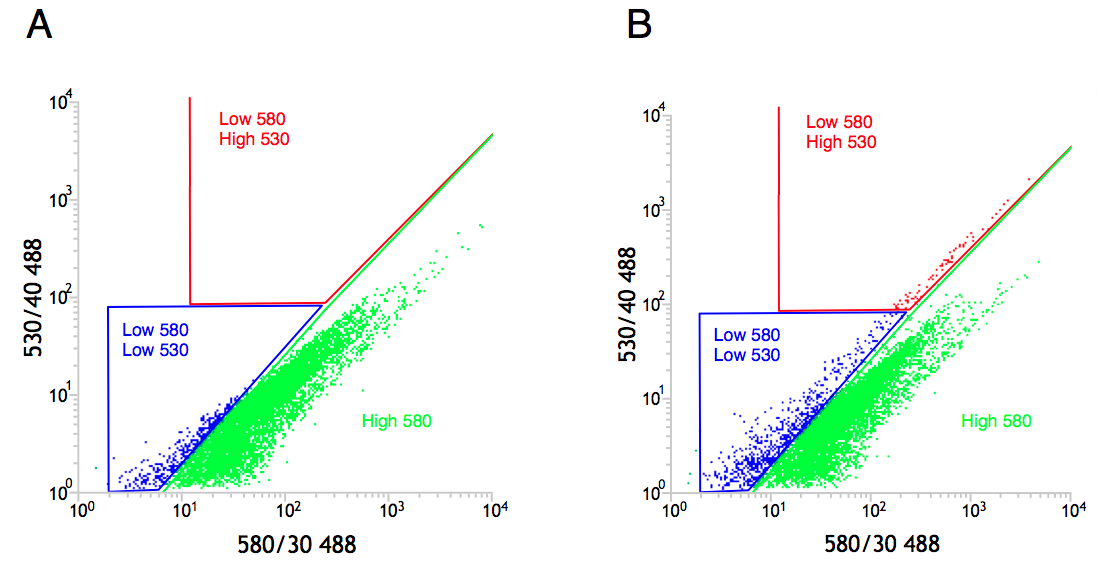

Supplement: Figure S2 — Fluorescence expression profiles for cells analyzed and sorted with FACS. (A,B) Dot plots of output from the 580/30 nm vs. 530/40 nm bandpass filters on the BD Influx, using a workspace derived from Grønlund et al. (2012). (A) Protoplasts generated from uninfected, 14-day-old ProDMR6::GFP seedlings, where cells were collected exclusively from the low 580/low 530 (GFP-negative) fate. (B) Protoplasts generated from ProDMR6::GFP inoculated with Hpa isolate Noks1, at 7 d.p.i., where cells were collected from both the low 580/low 530 (GFP-negative) and low 580/high 530 (GFP-positive, ~0.5%) gates. The high 580/low 530 gate represents cell debris. [file FigureS2.TIF]

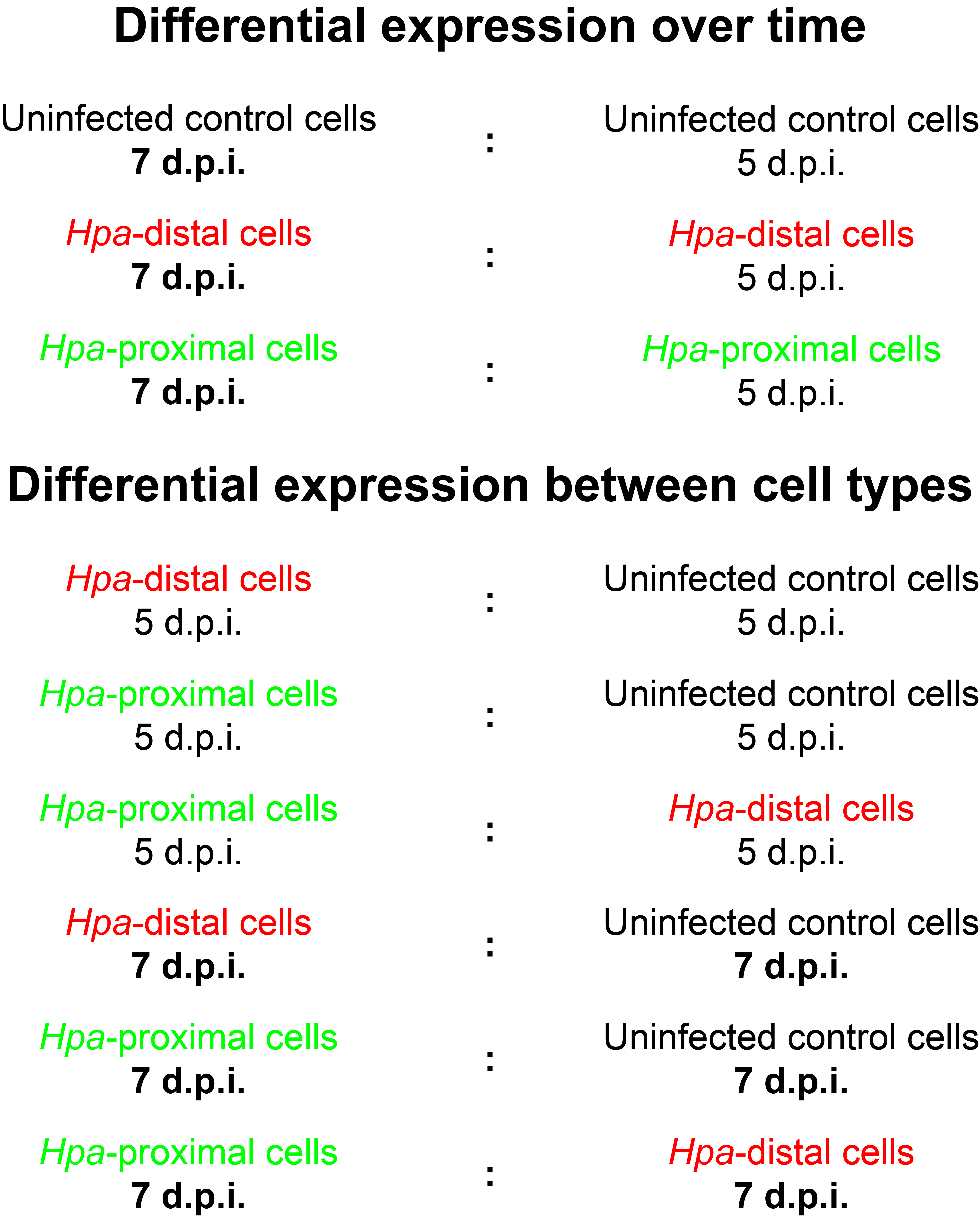

Supplement: Figure S3 — Pair-wise comparisons used to identify differentially expressed genes. Expression at 7 d.p.i. and 5 d.p.i. was compared for each cell type. Genes significantly differentially expressed in either Hpa-distal or Hpa-proximal cells, but not uninfected control cells, over time were considered as differentially expressed. To investigate differential expression between cell types, six comparisons were performed: Hpa-distal cells vs. uninfected control cells, Hpa-proximal cells vs. uninfected control cells, and Hpa-proximal cells vs. Hpa-distal cells, independently for each time point. [file FigureS3.TIF]
